# Application of chromosome microarray analysis and karyotyping in fetal cardiac abnormalities

**DOI:** 10.3389/fgene.2025.1611388

**Published:** 2025-06-25

**Authors:** Yun Guo, Xiaoqin Xin, Linju Zhou, Jungao Huang

**Affiliations:** ^1^ Department of Medical, Ganzhou Maternal and Child Health Hospital, Ganzhou, Jiangxi, China; ^2^ Department of Clinical Laboratory, Ganzhou People’s Hospital, Ganzhou, Jiangxi, China; ^3^ Department of Medical Genetic, Ganzhou Maternal and Child Health Hospital, Ganzhou, Jiangxi, China

**Keywords:** chromosome microarray analysis, karyotyping, fetal cardiac abnormalities, copy number variation (CNV), ultrasound abnormalities

## Abstract

**Objective:**

Chromosome microarray analysis (CMA) and karyotyping are two important genetic testing techniques used in prenatal diagnosis. This study aims to evaluate the value of chromosome microarray analysis and karyotyping in the diagnosis of fetal cardiac abnormalities, with particular focus on the detection of genomic copy number variations (CNVs).

**Methods:**

A retrospective analysis was conducted on 98 pregnant women diagnosed with fetal cardiac abnormalities through ultrasound between January 2022 and June 2024. Amniotic fluid samples from all participants were subjected to the analysis of karyotyping and Chromosome microarray analysis. The detection rates of both techniques in different types of fetal cardiac abnormalities were compared, and the outcomes of positive cases were followed up.

**Results:**

Of the 98 fetuses with cardiac abnormalities, 12 cases showed abnormal genetic results, with a detection rate of 12.24%. Karyotyping identified 5 cases of abnormalities (5.10%), while the chromosome microarray analysis detected 11 cases (11.22%). In the group with isolated cardiac abnormalities (76 cases) and the group with cardiac abnormalities combined with other ultrasound abnormalities (22 cases), karyotyping detected 3.95% (3/76) and 9.09% (2/22) of abnormalities, with no significant statistical difference (P > 0.05). Chromosome microarray analysis detected abnormalities in 6.58% (5/76) of the isolated cardiac abnormalities group and 27.27% (6/22) in the group with combined abnormalities, showing a significant statistical difference (P < 0.05). Of the 12 positive cases, four were live births, eight were terminations, and *postpartum* cardiac abnormalities were found in two live births during follow-up.

**Conclusion:**

Chromosome microarray analysis has a higher detection rate in fetuses with cardiac abnormalities than traditional chromosome karyotyping, especially when fetal cardiac abnormalities are combined with other ultrasound abnormalities. It is recommended for clinical use to improve the detection of genetic alterations.

## 1 Introduction

Birth defects are a leading cause of infant mortality and disability, with congenital heart defects accounting for 28% of all birth defects in China (Van der Linde et al., 2011; Kang et al., 2024; Han et al., 2024; Helm et al., 2016). These abnormalities can result in early embryonic loss or non-infectious infant mortality and are closely associated with chromosomal abnormalities and genomic copy number variations (CNVs). Fetal cardiac abnormalities are often detected through ultrasound, but the accuracy of ultrasound diagnoses can be affected by various factors such as the mother’s body condition and the technician’s experience (Meller et al., 2020). Therefore, it is important to use more sensitive diagnostic methods like chromosome microarray analysis technology and karyotyping to improve the accuracy of prenatal diagnosis. Karyotyping has long been considered the gold standard for detecting chromosomal abnormalities, but it is limited by its long turnaround time and lower sensitivity for small structural variations. In contrast, chromosome microarray analysis has gained popularity in recent years, can detect CNVs at a much higher resolution, especially for variants smaller than 5–10 Mb (Xia et al., 2018; Chen et al., 2021). CNV generally refers to duplication or deletion of genome fragments larger than 1kb, and this structural variation plays an important role in genome rearrangement, adaptive evolution, and disease progression. CNV is known to be widespread in the human genome, accounting for about 5%–10%. More than 38,000 CNVs have been identified, and related studies have shown that about 15% of genetic diseases are related to CNVs (Riggs et al., 2020a). However, although chromosome microarray analysis and karyotyping are important genetic detection techniques for fetuses with prenatal ultrasound abnormalities, there is sparse research on whether chromosome microarray analysis is superior to conventional karyotyping in fetal heart abnormalities. Therefore, we conducted a cohort study involving 98 fetuses with cardiac abnormalities to assess the detection rate of abnormalities by both techniques and differences in detection rates between chromosome microarray analysis and karyotyping in different group of fetal cardiac abnormalities to obtain their clinical utility as genetic diagnostic tools for prenatal fetal cardiac abnormalities.

## 2 Materials and methods

### 2.1 Study subjects

This retrospective study reviewed 98 pregnant women diagnosed with fetal cardiac abnormalities based on ultrasound examination at Ganzhou Maternal and Child Health Hospital between January 2022 and June 2024. Participants ranged in age from 19 to 51 years, with a mean age of 29.38 ± 4.71 years. The mean gestational age at prenatal diagnosis was 24^+3^ weeks (range 16–36^+6^ weeks). Inclusion criteria were: 1) fetal cardiac abnormalities detected by ultrasound, including Ventricular septal defect, pericardial effusion, tetralogy of Fallot, fetal echocardial cavity, arrhythmia, valvular regurgitation, abnormal pulmonary artery flow rate, aortic coarctation and other cardiac abnormalities; 2) pregnant women who underwent amniocentesis for chromosome karyotyping and chromosome microarray analysis. Exclusion criteria included: 1) family history of genetic disorders; 2) immune system diseases or severe psychiatric disorders in the pregnant women. Based on whether the fetal heart abnormality was accompanied by other ultrasonic abnormalities, participants with fetal heart abnormality was divided into isolated heart abnormality group and heart with other abnormalities group. Pregnant women and/or their families were informed of the risks of the surgery before amniocentesis, and the limitations and clinical significance of chromosome microarray analysis and chromosome karyotype testing were explained. The amniotic fluid was drawn after the puncture for follow-up testing. In addition, clinicians followed up by telephone and recorded clinical and pregnancy outcome data until 1 year after birth. Written informed consent was obtained from the pregnant women and/or their families.

### 2.2 Methods

#### 2.2.1 Karyotyping analysis

A total of 35 mL amniotic fluid was extracted through B-ultrasound assisted amniotic fluid puncture and divided into four 15 mL sterile centrifuge tubes, of which 20 mL was used for karyotype analysis. The amniotic fluid was inoculated in culture dishes and cultured for 8 days, the amniotic fluid cells were collected with pancreatic enzyme and colchicine, hypotonic treatment was performed according to standard procedures, and then prepared. The G-banding technique was used for analysis, and the karyotype was described according to the Nomenclature of Human Cytogenetics 2024 (ISCN 2024).

#### 2.2.2 Chromosome microarray analysis

The 15 mL amniotic fluid in the above process was centrifuged, the supernatant was poured, and the bottom was reserved for precipitation of 5 mL amniotic fluid. The next step was to split the precipitated cells to extract the DNA of the amniotic fluid cells, and the DNA concentration was determined (the DNA concentration should be ≥ 50 ng/μl), and about 250 ng of 5 μL was taken for further follow-up experiments. The DNA was digested by NspI enzyme to obtain the sticky terminal DNA fragments, and PCR amplification was performed using primers and specific connectors to obtain high-abundance genomic DNA (fragment size range: 150-2000bp). The dNTP, primers and enzymes were purified by magnetic beads, the PCR products were purified, and the DNA concentration was determined with requirements greater than 2.5 μg/μl, and the genomic DNA was further fragment with the length range of 25bp-125bp. Finally, the above small fragments were labeled by deoxynucleotide terminal transferase and hybridized with Affymetrix 750K chip, and the hybridization time was controlled within 16–18 h. After completing the hybridization, a washing process was carried out to remove the free DNA, and the final step was to scan the results using a scanner. Chromosomal microarray analysis (CMA) was performed using the Affymetrix CytoScan 750K array (Applied Biosystems, Affymetrix, Inc., Santa Clara, CA, USA) with the following criteria: (1) CNV detection: Size threshold >400 kb (spanning >50 consecutive markers) for both copy gains and losses; (2)ROH analysis: Homozygous regions >10 Mb were reported; (3) Probe coverage: All 22 autosomes and sex chromosomes were interrogated, with probe positions validated against the GRCh38 reference genome; (4) Excluded common variants: Population frequency ≥1% in DGV database (v2023.1); (5) American Society for Medical Genetics and Genomics (ACMG) 2020 guidelines were applied to determined pathogenicity classification of CNVs with annotations from DECIPHER, ClinVar, ClinGen Dosage Sensitivity Map, Pubmed, etc. (Riggs et al., 2020b). The interpretation of clinical significance of CNVs were divided into five categories (1)Likely benign CNV; (2) Benign CNV; (3) Variant of uncertain significance (VUS); (4) Pathogenic CNV; (5) Likely pathogenic CNV. Positive cases include individuals with variant of uncertain significance (VUS), likely pathogenic CNV, and pathogenic CNV.

### 2.3 Statistical analysis

Data were analyzed using SPSS 27.0 software. Continuous variables were expressed as mean ± standard deviation, while categorical variables were presented as percentages. Categorical variables were compared using Pearson’s chi-square test. For contingency tables meeting Cochran’s criteria (total sample size ≥40 and at least 1 cell with expected frequency 1 ≤ T < 5), Yates’ continuity correction was applied to mitigate type I error. When the total sample size (n < 40), or the expected frequency of any cell in the contingency table (T < 5), Fisher’s exact test was used. In order to verify the stability of the conclusion, multivariate logistic regression models were used to calculate odds ratios (ORs) and 95% confidence intervals (CIs). The covariates were not adjusted in the crude model 1. The adjusted covariates in Model two include age, gestational weeks, external hospitals (no/yes), number of pregnancies and parity. Statistical significance was defined as a two-tailed P-value <0.05.

## 3 Results

### 3.1 Overall result

Among the 98 fetal cardiac abnormalities, 12 cases were positive for a genetic abnormality and the detection rate was 12.24% (12/98). Among them, 11 cases were found by chromosome microarray analysis and the detection rate was 11.22% (11/98), and 5 cases were found by karyotyping, and the detection rate was 5.10% (5/98), and a total of 4 cases were detected by both techniques (Shown in Tables 1, 3, 5).

**TABLE 1:** Comparison of Chromosomal Abnormality Rates Detected by Karyotyping analysis among Different Types of Fetal Cardiac Anomalies.

Group	Total Cases	Detected Abnormalities (cases)	Detection Rate (%)	χ^2^	*P*
Isolated Cardiac Anomaly Group	76	3	3.95	0.173	P = 0.678
Cardiac Anomalies with Other Abnormalities Group	22	2	9.09		
Total	98	5	5.10		

### 3.2 Karyotyping analysis results for isolated cardiac anomalies vs. cardiac anomalies combined with other abnormalities

In the group with isolated cardiac anomalies, there were 76 cases observed, with three identified as having chromosomal abnormalities, resulting in an abnormality rate of 3.95% (3/76). In the group of cardiac anomalies combined with other abnormalities, a total of 22 cases were analyzed, leading to the identification of chromosomal abnormalities in 2 cases, which corresponds to an abnormality rate of 9.09% (2/22). No statistically significant difference was found between the detection rates of the two groups (P > 0.05) (Shown in Table 1). Table 2 explored the relationship between cardiac anomaly type and the results of karyotyping analysis using univariate and multivariate logistic regression analyses and the relationship did not found in crude model one and adjusted model 2.

**TABLE 2:** Associations between cardiac anomaly type and the results of karyotyping analysis.

Cardiac anomaly type	Event (%)	Crude Mode l		Model 2	
		OR (95% CI)	p value	OR (95% CI)	p value
Isolated Cardiac Anomaly	3 (3.95)	1(Ref)		1(Ref)	
Cardiac Anomalies with Other Abnormalities	2 (9.09)	2.43 (0.38–15.57)	0.348	2.74 (0.18–41.17)	0.466

Ref: reference; OR: odds ratio; CI: confidence interval.

Model 1: adjusted for none.

Model 2: adjusted for age, gestational weeks, external hospitals (no/yes), number of pregnancies and parity.

### 3.3 Chromosome microarray analysis results for isolated cardiac anomalies vs. cardiac anomalies combined with other abnormalities

In the isolated cardiac anomaly group, chromosome microarray analysis detected abnormalities in 5 cases, accounting for 6.58% (5/76). In the group with cardiac anomalies combined with other abnormalities, a total of 6 cases were identified as having abnormalities, resulting in a detection rate of 27.27% (6/22). Statistical analysis indicated a significant difference between two groups (P < 0.05) (Shown in Table 3). Table 4 shown there was a significant association between cardiac anomaly type and the results of chromosomal microarray analysis in crude model one and the association remained robusted in model 2. The potential genetic variation risk of cardiac anomalies with other abnormalities had increased by 6.95 times compared to the isolated cardiac anomaly (OR = 7.95, 95% CI:1.43–44.18, P = 0.018).

**TABLE 3:** Comparison of Chromosomal Abnormality Rates Detected by Chromosome microarray analysis among Different Types of Fetal Cardiac Anomalies.

Group	Total Cases	Detected Abnormalities (cases)	Detection Rate (%)	χ^2^	*P*
Isolated Cardiac Anomaly Group	76	5	6.58	5.402	P = 0.020
Cardiac Anomalies with Other Abnormalities Group	22	6	27.27		
Total	98	11	11.22		

**TABLE 4:** Associations between cardiac anomaly type and the results of chromosomal microarray analysis.

Cardiac anomaly type	Event (%)	Crude Mode l		Model 2	
		OR (95% CI)	p value	OR (95% CI)	p value
Isolated Cardiac Anomaly	5 (6.58)	1(Ref)		1(Ref)	
Cardiac Anomalies with Other Abnormalities	6 (27.27)	5.32 (1.44–19.64)	0.012	7.95 (1.43–44.18)	0.018

Ref: reference; OR: odds ratio; CI: confidence interval.

Model 1: adjusted for none.

Model 2: adjusted for age, gestational weeks, external hospitals (no/yes), number of pregnancies and parity.

### 3.4 Follow-up outcomes of chromosomal abnormalities in fetuses with cardiac anomalies

Among the 12 samples from fetuses with ultrasound-detected cardiac anomalies, chromosomal aneuploidy was identified in 5 cases. Specifically, this included 2 cases of Trisomy 18, 1 case of Trisomy 21, 1 case of 47,XXX karyotype, and 1 case of an interstitial inversion of chromosome 20. Additionally, 7 cases were noted to be associated with CNVs, with three exhibiting pathogenic CNVs, including 2 cases of microdeletion at 22q11.21 and 1 case of microdeletion at 15q11.1, while 4 cases showed clinically insignificant CNVs. Follow-up outcomes from the 12 positive cases revealed that four resulted in live births, of which 2 neonates continued to show cardiac anomalies upon later examination, while the other two did not exhibit any abnormalities. The pregnancies of the remaining 8 cases were terminated (Shown in Table 5).

**TABLE 5:** Chromosomal abnormalities and follow-up outcomes in 12 fetuses with cardiac anomalies.

No.	Age	Gestational week	Ultrasound abnormal findings	Chromosomal abnormality results	Clinical significance of CNV	The identified method	Follow-up outcomes
1	21	25 + 1	Ventricular septal defect, lymphatic cyst	arr [GRCh38] 15q11.1 (22,578,121-23,096,049)x1	Pathogenic	CMA	Full-term vaginal delivery, 1 female, ventricular septal defect, heart murmur
2	30	23 + 6	“C” type vascular ring malformation, right subclavian artery nerve anomaly	arr [GRCh38] 22q11.21 (18,929,330-21,110,475)×1	Pathogenic	CMA	Pregnancy termination
3	31	24	Multiple cardiac malformations	arr [GRCh38] 22q11.21 (18,153,983-21,110,475)×1	Pathogenic	CMA	Pregnancy termination
4	27	30 + 6	Pericardial effusion, mild tricuspid regurgitation, arachnoid cyst	arr [GRCh38] 22q11.23 (23,579,020-24,884,874)×3	VUS	CMA	Full-term vaginal delivery, 1 male, no abnormalities observed
5	37	19 + 6	Ventricular septal defect, single umbilical artery	arr [GRCh38] 22q11.22 (21,946,280-22,224,671)×1	VUS	CMA	Pregnancy termination
6	33	27+	Tricuspid regurgitation, pericardial effusion	arr [GRCh38] 1p36.21p35.3 (40,844,761-54,566,668)x2 hmz arr [GRCh38] 7p14.3p12.1 (32,190,335-50,447,095)x2 hmz	VUS	CMA	Pregnancy termination
7	33	25 + 3	Tetralogy of Fallot	arr [GRCh38] 7q33q36.3 (137,588,008-156,073,667)x2 hmz	VUS	CMA	Pregnancy termination
8	32	20 + 5	Ventricular septal defect	47,XN,+21	—	CMA and karyotyping	Pregnancy termination
9	30	18	NT thickening, bilateral choroid cysts, ventricular septal defect	47,XN,+18	—	CMA and karyotyping	Pregnancy termination
10	37	19 + 3	Ventricular septal defect, right choroid cyst	47,XN,+18	—	CMA and karyotyping	Pregnancy termination
11	30	30 + 3	Ventricular septal defect	47,XXX	—	CMA and karyotyping	Full-term cesarean section, 1 female, no abnormalities observed
12	26	24	Ventricular septal defect	46,XN,inv (20) (p12∼q12)	—	Karyotyping	Full-term cesarean section, 1 male, ventricular septal defect

CMA: chromosomal microarray analysis; CNV: copy number variation; VUS:variant of uncertain significance.

## 4 Discussion

Fetuses with cardiac defects represent one of the most prevalent types of birth defects, impacting eight to nine out of every 1,000 live births and occurring in 10% of spontaneous abortions (Menahem and Meagher, 2021; Fahed et al., 2013). The issue of cardiac abnormalities in fetuses is intricate, as it encompasses both genetic and environmental factors (Pei et al., 2017). After the identification of fetal cardiac anomalies through ultrasound, it becomes imperative to further assess the potential for chromosomal abnormalities, the presence of extracardiac anomalies, and the specific type of heart defect. Such evaluations play a vital role in guiding informed decisions regarding the continuation of pregnancy. Ultrasound remains the primary screening tool for fetal cardiac anomalies due to its safety, non-invasive nature, and ease of use. However, factors such as maternal health conditions, operator expertise, and technological limitations can affect its accuracy (Fuchs et al., 2013).

In our study, we employed both karyotyping and chromosome microarray analysis to investigate fetuses diagnosed with cardiac abnormalities via ultrasound. Our findings revealed that chromosomal abnormalities were detected in 12.24% of the overall cases. Notably, chromosome microarray analysis demonstrated a markedly higher detection rate of 11.22% compared to traditional chromosomal karyotyping, which revealed a detection rate of only 5.10%. The overall abnormal detection rate of CMA for cardiac malformations was close to 15.3% (98/642) reported by Lu et al. in a cohort (Lu et al., 2024). Moreover, a recent meta-analysis conducted by Wang H et al. concluded that cardiac defects frequently coexisted with genomic anomalies and when results of traditional karyotyping were negative, CMA became essential for identifying submicroscopic genomic anomalies, particularly in complex cardiac anomalies accompanied by septal abnormalities (Wang et al., 2023). Interestingly, the detection rate of traditional chromosomal karyotyping did not show a statistically significant difference between the isolated cardiac abnormality group (3.95%) and the group with combined abnormalities (9.09%) (P > 0.05). However, the application of chromosome microarray analysis revealed a significant difference, detecting abnormalities in 6.58% of the isolated cardiac anomaly group compared to 27.27% in the combined anomaly group (P < 0.05). This finding underscored the importance of considering additional anomalies when a fetal cardiac defect was identified through ultrasound. It aligned with earlier research by Fortin et al. (2014), who also emphasized the relevance of comprehensive genomic evaluation in fetuses presenting with multiple congenital anomalies, thus highlighting a tailored approach to prenatal diagnosis (Fortin et al., 2024). In our follow-up of the 12 cases identified as positive for chromosomal abnormalities, we noted that five fetuses presented with aneuploidy, including trisomy 18, trisomy 21, and 47,XXX, along with a 20q interstitial inversion. These findings were consistent with the literature indicating that aneuploidies were commonly associated with fetuses with major heart defects (Hartman et al., 2011). The chromosome 20 inversion likely represented a balanced rearrangement with uncertain clinical significance. The CMA detected no copy number changes at breakpoints and according to ACMG guidelines, inversions without gene disruption were generally considered likely benign. Additionally, seven cases exhibited copy number variations (CNVs), three of which were pathogenic including two instances of microdeletion at 22q11.21, a region frequently implicated in cardiac anomalies as noted by Mustillo et al. (2023). Our follow-up evaluation revealed that four live births occurred, with two demonstrating postnatal cardiac abnormalities, while the other two displayed no observable anomalies. Cases with postnatal cardiac defects despite normal CMA results highlighted inherent limitations of CMA technology. CMA detected only large copy number variations and missed single-nucleotide variants, small indels, balanced rearrangements, and epigenetic modifications that contributed to ∼30% of congenital heart defects (Liao et al., 2014; Cai et al., 2020), which may emphasize the need for: 1) Combined exome sequencing to identify small coding variants, 2) Continued postnatal surveillance given potential oligogenic/environmental interactions. These follow-up outcomes were crucial in assessing the effectiveness of prenatal diagnoses, particularly in validating the presence of chromosomal abnormalities.

Despite the insights provided by this study, it is essential to recognize its limitations, including a relatively small sample size. However, the total detection rate of fetal cardiac abnormalities by CMA was close to that reported by Lu et al. in a relatively large population (Lu et al., 2024). Meanwhile, We were aware that some samples lacked trio-based testing (samples from parents), which was important for CNVs of the VUS type, especially. However, this was mainly due to considering practical factors during the clinical process. The price of a single CMA sample was very expensive in underdeveloped areas, and if both parents’ samples were used for CMA together, it would bring a heavy economic burden to the pregnant woman’s family. Besides, absence of validation of CNVs by orthogonal methods had not been implemented and it had to be admitted that for CNVs <100 kb (such as 22q11.2 microdeletions), supplementary MLPA validation was still required. And other methods such as FISH, qPCR or ddPCR validation were sometimes also necessary. Lastly, a brief follow-up period should also be noted. Although congenital heart diseases could be largely screened clinically in the early stage, it was also necessary to further prolong the period for neuro-related diseases. Therefore, further investigations with larger sample sizes and extended follow-up durations are necessary to enhance our understanding of fetal cardiac anomalies and their associations.

In conclusion, this study advocates for chromosome microarray analysis as the preferred diagnostic method for fetal cardiac abnormalities detected via ultrasound, particularly in the presence of accompanying congenital anomalies. Its superior detection rate for CNVs, compared to traditional chromosomal karyotyping, provides a higher resolution for prenatal diagnoses, thereby facilitating better guidance for reproductive decision-making and interventions.

## Data Availability

The original contributions presented in the study are included in the article/supplementary material, further inquiries can be directed to the corresponding author.

## References

[B1] Cai M. Lin N. Su L. Wu X. Xie X. Li Y. (2020). Detection of copy number disorders associated with congenital anomalies of the kidney and urinary tract in fetuses via single nucleotide polymorphism arrays. J. Clin. Lab. Anal. 34 (1), e23025. 10.1002/jcla.23025 31506986 PMC6977156

[B50] Chen Y Lai Y Xu F Qin H Tang Y Huang X (2021). The application of expanded noninvasive prenatal screening for genome-wide chromosomal abnormalities and genetic counseling. J. Matern. Fetal. Neonatal Med. 34 (16), 2710–2716. 10.1080/14767058.2021.1907333 33938369

[B2] Fahed A. C. Gelb B. D. Seidman J. G. Seidman C. E. (2013). Genetics of congenital heart disease: the glass half empty. Circ. Res. 112 (4), 707–720. 10.1161/CIRCRESAHA.112.300853 23410880 PMC3827691

[B3] Fortin O. Mulkey S. B. Fraser J. L. (2024). Advancing fetal diagnosis and prognostication using comprehensive prenatal phenotyping and genetic testing. Pediatr. Res. 10.1038/s41390-024-03343-9 38937640

[B4] Fuchs F. Houllier M. Voulgaropoulos A. Levaillant J. M. Colmant C. Bouyer J. (2013). Factors affecting feasibility and quality of second-trimester ultrasound scans in obese pregnant women. Ultrasound Obstet. Gynecol. 41 (1), 40–46. 10.1002/uog.12311 23023941

[B5] Han J. Zhang Y. Liu Y. Liu J. Zhang Y. Wang K. (2024). Parental smoking and the risk of birth defects in offspring in China: a systematic review and meta-analysis. Birth Defects Res. 116 (12), e2422. 10.1002/bdr2.2422 39648656

[B6] Hartman R. J. Rasmussen S. A. Botto L. D. Riehle-Colarusso T. Martin C. L. Cragan J. D. (2011). The contribution of chromosomal abnormalities to congenital heart defects: a population-based study. Pediatr. Cardiol. 32 (8), 1147–1157. 10.1007/s00246-011-0034-5 21728077

[B52] Helm B. M. Freeze S. L. (2016). Genetic Evaluation and Use of Chromosome Microarray in Patients with Isolated Heart Defects: Benefits and Challenges of a New Model in Cardiovascular Care. Front. Cardiovasc. Med. 3, 19. 10.3389/fcvm.2016.00019 27379245 PMC4905945

[B7] Kang L. Y. Guo Z. R. Shang W. J. Cao G. Y. Zhang Y. P. Wang Q. M. (2024). Perinatal prevalence of birth defects in the Mainland of China, 2000-2021: a systematic review and meta-analysis. World J. Pediatr. 20 (7), 669–681. 10.1007/s12519-023-00786-8 38340146

[B8] Liao C. Fu F. Li R. Xie G. E. Zhang Y. L. Li J. (2014). Implementation of high-resolution SNP arrays in the investigation of fetuses with ultrasound malformations: 5 years of clinical experience. Clin. Genet. 86 (3), 264–269. 10.1111/cge.12271 24000829

[B9] Lu Q. Luo L. Zeng B. Luo H. Wang X. Qiu L. (2024). Prenatal chromosomal microarray analysis in a large Chinese cohort of fetuses with congenital heart defects: a single center study. Orphanet J. Rare Dis. 19 (1), 307. 10.1186/s13023-024-03317-4 39175064 PMC11342572

[B10] Menahem S. Meagher S. (2021). Early detection of congenital heart disease: the contribution of fetal ultrasound and newborn screening. Paediatr. and Child Health 57 (3), 323–327. 10.1111/jpc.15355 33529483

[B51] Meller C. H Grinenco S. Aiello H. Córdoba A. Sáenz-Tejeira M. M. Marantz P. (2020). Congenital heart disease, prenatal diagnosis and management. Arch. Argent. Pediatr. 118 (2), e149–e161. 10.5546/aap.2020.eng.e149 32199055

[B11] Mustillo P. J. Sullivan K. E. Chinn I. K. Notarangelo L. D. Haddad E. Davies E. G. (2023). Clinical practice guidelines for the immunological management of chromosome 22q11.2 deletion syndrome and other defects in thymic development. J. Clin. Immunol. 43 (2), 247–270. 10.1007/s10875-022-01418-y 36648576 PMC9892161

[B12] Pei L. Kang Y. Zhao Y. Yan H. (2017). Prevalence and risk factors of congenital heart defects among live births: a population-based cross-sectional survey in Shaanxi province, Northwestern China. BMC Pediatr. 17 (1), 18. 10.1186/s12887-017-0784-1 28086762 PMC5237335

[B13] Riggs E. R. Andersen E. F. Cherry A. M. Kantarci S. Kearney H. Patel A. (2020a). Technical standards for the interpretation and reporting of constitutional copy-number variants: a joint consensus recommendation of the American College of Medical Genetics and Genomics (ACMG) and the Clinical Genome Resource (ClinGen). Genet. Med. 22 (2), 245–257. 10.1038/s41436-019-068-8 31690835 PMC7313390

[B14] Riggs E. R. Andersen E. F. Cherry A. M. Kantarci S. Kearney H. Patel A. (2020b). Technical standards for the interpretation and reporting of constitutional copy-number variants: a joint consensus recommendation of the American College of Medical Genetics and Genomics (ACMG) and the Clinical Genome Resource (ClinGen). Genet. Med. 22 (2), 245–257. 10.1038/s41436-019-0686-8 31690835 PMC7313390

[B15] Van der Linde D. Konings E. E. M. Slager M. A. Witsenburg M. Helbing W. A. Takkenberg J. J. M. (2011). Birth prevalence of congenital heart disease worldwide: a systematic review and meta-analysis. J. Am. Coll. Cardiol. 58 (21), 2241–2247. 10.1016/j.jacc.2011.08.025 22078432

[B17] Wang H. Lin X. Lyu G. He S. Dong B. Yang Y. (2023). Chromosomal abnormalities in fetuses with congenital heart disease: a meta-analysis. Arch. Gynecol. Obstet. 308 (3), 797–811. 10.1007/s00404-023-06910-3 36609702

[B53] Xia Y. Yang Y. Huang S. Wu Y. Li P. Zhuang J. (2018). Clinical application of chromosomal microarray analysis for the prenatal diagnosis of chromosomal abnormalities and copy number variations in fetuses with congenital heart disease. Prenat. Diagn. 38 (6), 406–413. 10.1002/pd.5249 29573438

